# MMP-2 salivary activity in type 2 diabetes mellitus patients

**DOI:** 10.1186/s13098-019-0510-2

**Published:** 2019-12-30

**Authors:** Juan Antonio Arreguin-Cano, Brenda Ayerdi-Nájera, Arvey Tacuba-Saavedra, Napoleón Navarro-Tito, Alfonso Dávalos-Martínez, Abel Emigdio-Vargas, Elia Barrera-Rodríguez, Nubia Blanco-García, Gloria Gutiérrez-Venegas, Elías Ventura-Molina, Gladys León-Dorantes

**Affiliations:** 1 Unit of Clinical and Epidemiological Innovation of the State of Guerrero, Secretary of Health of the State of Guerrero, Av. Juan R. Escudero No. 158 Col. C.D. Renacimiento, 39715 Acapulco, Guerrero Mexico; 2 Laboratory of Biochemistry, School of Dentistry, National Autonomous University of Mexico, Mexico City, Mexico; 3 Laboratory of Cellular Biology of Cancer, School of Chemical Sciences-Biological, University Autonomy of Guerrero, Chilpancingo, Guerrero Mexico; 4 Intelligent Computing Laboratory, Computer Research Center, Polytechnic Institute in Computing, Mexico City, Mexico

**Keywords:** Diabetes mellitus type 2, MMP-2, Periodontitis, TIMP-1

## Abstract

**Background:**

Type 2 diabetes mellitus (T2DM) and periodontitis are chronic inflammatory diseases with a bidirectional relationship. The uncontrolled levels of glucose in T2DM patients change the pathophysiology and balance of inflammatory mediators. Matrix Metalloproteinase-2 (MMP-2) is a zinc-dependent endopeptidase that is responsible for tissue remodeling and degradation of the extracellular matrix in periodontal tissue. Therefore, the uncontrolled levels of glucose in T2DM could lead to an imbalance in MMP-2 activity in saliva, favoring the development of periodontitis.

**Methods:**

Ninety-seven T2DM patients from Hospital Dr. Donato Alarcon were included in the study. Following clinical examination, the patients were classified into four groups according to the presence and degree of periodontal disease and glycemic control. Blood and whole saliva samples (WSS) were collected from each patient. Blood samples were used for Hba1c and polymorphonuclear cells count determination, while WSS were used to determine MMP-2 activity, TIMP-1 and nitrite. MMP-2 activity was determined by zymography. TIMP-1 were determined by Western blotting, and nitric oxide (NO) levels were determined by the Griess method.

**Results:**

Of the 97 patients with T2DM, 66 had periodontitis of different severities: 18 patients had mild periodontitis, 15 had moderate and 33 had severe. Salivary MMP-2 activity, HbA1c and TIMP-1 were positively correlated with the severity of periodontitis. On the other hand, the increase in HbA1c was negatively correlated with MMP-2 activity and quantity of TIMP-1 but was positively correlated with nitrite levels.

**Conclusions:**

T2DM with glycemic uncontrol conditions, distinct clinical alterations in periodontal tissue were identified, including a decrease in the gingival redness, increased the clinical attachment loss and imbalance of MMP-2/TIMP-1, as the possible causes of disorders promoting the progression of periodontitis. Accelerated periodontitis development with poor glycemic uncontrol likely results from the altered response of host defenses and decreased activity of polymorphonuclear cells. Taken together, these findings identify MMP-2 as a promising molecular market for periodontitis.

## Background

Periodontitis is a chronic infection-induced inflammatory disease that causes tooth loss and is also considered a modifying factor of systemic health [1, 2]. Type 2 diabetes mellitus (T2DM) is a metabolic disorder characterized by high levels of blood glucose resulting from altered insulin secretion or action [3]. Periodontitis and T2DM are chronic, multifactorial and highly prevalent diseases [4, 5]. T2DM is considered a major risk factor for periodontitis [4]. The mechanisms that link T2DM and periodontitis involve immune response, inflammation, neutrophil activity, and cytokine release [6]. Moreover, T2DM increases inflammation in periodontal tissues, with high levels of inflammatory mediators such as interleukin-1β (IL-1β), tumor necrosis factor-α (TNFα), nitrites and increased matrix metalloproteinases (MMPs) activity [7, 8]. Furthermore, an increase in the levels of glucose in T2DM patients decreases the immune response of macrophages and reduces the production of collagen by fibroblasts, resulting in delayed tissue recovery and changing the etiopathology of different diseases [4, 8].

MMPs are zinc-dependent endopeptidases that are responsible for tissue remodeling and degradation of the extracellular matrix (ECM) [9, 10]. MMPs are involved in crucial events in tissue remodeling both in physiological processes such as reproduction and embryonic development, as well as in pathological conditions such as arthritis [11] and cardiovascular diseases [12]. MMPs regulate the activity of several non-ECM bioactive substrates that affect different cellular functions [13]. These substrates are proinflammatory and anti-inflammatory cytokines, chemokines, growth factors, serpins, serum components, complement components, and cell signaling molecules that modify immune responses [14]. The main MMPs present in the oral cavity are interstitial collagenases (MMP-1 and MMP-8), stromelysins (MMP-3 and MMP-10) and collagenases (MMP-2 and MMP-9). MMP-2, MMP-8 and MMP-9 are MMPs in saliva, with higher protease activity playing an important role in the degradation of periodontal tissues [9, 10].

MMP-2 cleaves type IV collagen and native type I collagen, which are synthesized by fibroblasts, endothelial cells and osteoblasts and are abundant components of gingival connective tissue and alveolar bone [15]. The main sources of MMP-2 found in saliva are polymorphonuclear leukocytes [16]. MMP-2 activity is regulated by tissue inhibitors of matrix metalloproteinases (TIMPs) [15], mainly TIMP-1, which is an endogenous inhibitor of MMP-2 that is produced by periodontal cells, macrophages, and monocytes [17].

During periodontal tissue inflammation, overexpression of MMPs leads to an increase in MMP levels in biological fluids such as saliva and gingival crevicular fluid [18, 19]. Previous studies on this topic have found higher levels of MMP-2 in saliva from patients with periodontitis [20, 21]. Other studies showed contradictory information, finding reduced levels of MMP-2 in patients with periodontitis [22, 23]. Moreover, clinical correlation studies suggest that high circulating MMP-2 levels may correlate with the severity of periodontitis in T2DM [24]. Nevertheless, the relationship between uncontrolled glycemia and the gelatinolytic activity of MMP-2 in the saliva of T2DM patients with periodontitis is unknown [25–27]. The main purpose of this study was to describe and compare the levels of salivary MMP-2 activity in uncontrolled glycemic T2DM patients.

## Materials and methods

### Study population

Patients with Type 2 diabetes mellitus attending the Diabetes Clinic at “Hospital Dr. Donato G. Alarcón” in Acapulco Guerrero, México, were enrolled in the study from October to December 2017. All participants provided written informed consent. T2DM patients were included in the study if the following criteria were met: absence of systemic disease other than T2DM that might influence the course of periodontal disease such as human immunodeficiency virus/acquired immunodeficiency syndrome (HIV/AIDS) or autoimmune diseases; no antibiotic therapy or antioxidant drugs at least 3 months prior to the sampling; no history of tobacco smoking or alcoholism; no prior periodontal therapy; and having at least 20 natural teeth (excluding third molars). Pregnant or lactating woman were excluded from the study. This observational cross-sectional study was approved by the Ethics Committee of Guerrero’s Secretariat of Health (Research Committee of the Guerrero State Health Services, Number 03301117).

### Saliva sample collection

Saliva samples of the patients included in this study met the following criteria: no eating, drinking or teeth brushing in the morning before sample collection and prior to clinical examination. Whole saliva samples (WSS) were collected in sterile tubes by paraffin stimulation and after carefully rinsing their mouths with 10 mL of distilled water to eliminate exfoliated cells. Approximately 5 mL of WSS was immediately centrifuged (700×*g* for 15 min at 4 °C) to remove cells without lysis. The supernatant was collected and centrifuged at 12,000×*g* for 10 min at 4 °C to remove all suspended insoluble debris. Samples from each participant were collected in one session between 9 and 11 a.m. The samples were stored at − 80 °C without thawing until analysis to preserve the integrity of MMP activity.

### Glycated hemoglobin percentage

After twelve hours of fasting, capillary blood samples were collected from patients to obtain HbA1c using the A1CNow + kit [28]. Patients were classified as having good (HbA1c ≤ 5.9%), regular (HBA1c 6.0–7.9%) or poor control (HBA1c > 8%) based on the parameters of the American Diabetes Association [29].

### Clinical monitoring

Clinical oral examinations were carried out by two calibrated dentists at the Unit of Clinical Innovation and Epidemiology of the State of Guerrero (UICyEEG), with a Kappa coefficient greater than or equal to 0.85. Clinical measurements were taken at six sites per tooth (mesio-buccal, buccal, disto-buccal, disto-lingual, lingual, and mesio-lingual) for teeth present, excluding the third molars, (a maximum of 168 sites per person) following the method described by Haffajee [30].

Clinical assessment included plaque accumulation (0/1; undetected/detected), overt gingivitis (0/1), bleeding on probing (0/1), suppuration (0/1), probing pocket depth and probing attachment level. Pocket depth and attachment level measurements were taken twice by the same examiner at each visit, and the average of the two measurements was recorded to the nearest millimeter using a North Carolina periodontal probe (Hu-Friedy, Chicago, IL). All measurements for a given subject were performed by the same examiner at each visit. The clinical characteristics of the 97 patients are presented in Table 1.

**Table 1:** Clinical characteristics and parameters of the subject group (N = 97)

		Range
Age (years)	58.97 ± 10.7	35–79
Gender (female/male)	72/25	
Missing teeth	5.21 ± 2.7	0–8
Periodontal health		
Healthy	31 (32%)	
Mild periodontitis	18 (19%)	
Moderate periodontitis	15 (15%)	
Severe periodontitis	33 (34%)	

Age, missing teeth, are show as mean ± SD and range

Periodontal status are show as percentage of total

### Periodontitis classification

Patients were grouped based on their periodontal health status into three groups according to the parameters of the American Academy Periodontology (AAP): mild (> 3 and < 5 mm probing depths and 1 to 2 mm of clinical attachment loss), moderate (> 5 and < 7 mm probing depths and 3 to 4 mm of clinical attachment loss) and severe (> 7 probing depths and > 5 mm of clinical attachment loss). Periodontally healthy individuals had fewer than three sites with a 3 mm attachment level and no sites with a 5 mm attachment level.

### Zymography

MMP-2 activity in WSS was assayed by gelatin zymography as previously described [31–33]. The gelatinolytic activity was detected as clear bands with a dark background corresponding to nondegraded gelatin. Supernatants from cultured MCF7 cells were used as a positive control. The lysis bands were quantified using the software Fiji (National Institute of Health, Bethesda, MD, USA). Resulting values were used as relative arbitrary units for MMP activity.

### Western blot analysis

Ten micrograms of whole protein extracts were separated by 10% SDS-PAGE, transferred to PVDF membranes and then incubated with anti-TIMP-1 (1:1000; sc-365905, Santa Cruz) primary antibody. Subsequently, anti-mouse secondary antibody (1:500; sc-2005, Santa Cruz) was added for 30 min in a humidified chamber in the dark at room temperature. The membranes were developed using an enhanced chemiluminescence detection system from Bio-Rad (Hercules, CA, USA). All experiments were performed in triplicate and were analyzed with ImageJ (National Institutes of Health) [34]. Band intensities were quantified using the software Fiji (National Institute of Health, Bethesda, MD, USA). Resulting values were used as relative arbitrary units for TIMP-1.

### Measurement of nitric oxide production

The release of nitrite, a stable product of NO in aqueous medium, was measured using the Griess method. Briefly, 100 μL of 1% sulfanilamide in 5% phosphoric acid was added to 100 μL of WWS. After 10 min of incubation at 23 °C, the absorbance at 550 nm was determined. The micromolar concentration of nitrite was calculated using a standard curve composed of sodium nitrite as a reference compound [35].

### Polymorphonuclear cell counts in peripheral blood samples

Peripheral blood samples were collected from each patient. Blood smears were prepared and stained by Wright-Giemsa (Beijing Midwest Technology Co, Ltd.) for 10 min. The counts were analyzed according to a laboratory procedure using a microscope. The first 100 white cells were counted and classified as mononuclear and polymorphonuclear cells [36].

### Statistical analysis

Differences in MMP-2 activity, TIMP-1 and the release of nitrite were performed by determining the integrated optical density of each sample between patient groups. A Kruskal–Wallis one-way ANOVA with Mann–Whitney U test was performed. Values of p < 0.05 were considered statistically significant.

HbA1c, polymorphonuclear cell counts, plaque, gingival redness, bleeding on probing, suppuration, probing pocket depth and clinical attachment loss were assessed by repeated-measures analysis of variance (ANOVA) followed by Bonferroni test (Prism 5, GraphPad software Inc., San Diego, USA) Values of p < 0.05 were considered as statically significant. Data are presented as the mean ± standard deviation of the mean (SD).

## Results

### Clinical parameters

Ninety-seven T2DM patients were included in the study, 72 female and 25 male, and their mean age was 58.97 (± 10.7 SD). Out of the 97 patients, 66 (68.04%) had periodontitis, and 31 (32%) did not. In those with periodontitis, 18 (19%) were mild, 15 (15%) were moderate, and 33 (34%) were severe (Table 1). Patients were grouped based on their periodontal status (periodontally healthy, mild periodontitis, moderate periodontitis and severe periodontitis) based on the AAP classification. A significant increase in gingival redness (p < 0.016), pocket probing depth (p < 0.02), and clinical attachment loss (p < 0.01) was observed in patients with severe periodontitis compared to those of periodontally healthy individuals (Table 2). When grouped according to their glycated hemoglobin percentage (good control ≤ 5.9%, regular control 6.0–7.9% and poor control > 8% of HbA1c), we observed a significant decrease in gingival redness and an increase in clinical attachment loss in patients with poor glycemic control compared to those with good glycemic control (p < 0.01) (Table 3).

**Table 2:** Clinical characteristics of periodontal status

	Healthy	Mild P.	Moderate P.	Severe P.	p-value
	n = 31	n = 18	n = 15	n = 33	
Plaque (%)	94.39 ± 19.85	87.74 ± 30.14	100 ± 0	90.43 ± 27.63	0.79
Gingival redness (%)	4.68 ± 18.81	5.68 ± 11.58	6.38 ± 11.47	14.68 ± 21.04	< 0.016*
Bleeding on probing (%)	17.72 ± 22.31	19.15 ± 19.17	27.05 ± 30.87	19.93 ± 24.95	0.72
Suppuration (%)	0	0	0.3 ± 1.011	0.43 ± 1.15	0.79
Pocket probing depth (mm)	1.47 ± 0.326	1.52 ± 0.57	1.95 ± 0.55	2.32 ± 0.72	< 0.02*
Clinical attachment loss (mm)	1.12 ± 0.656	1.7 ± 0.8	2.4 ± 0.66	3.4 ± 1.8	< 0.01*

Plaque, gingival redness, bleeding on probing, suppuration, pocket probing depth, clinical attachment loss are show as mean ± SD; all presented

* p values are based on repeated measures ANOVA

**Table 3:** Clinical characteristics periodontal

	HbA1c ≤ 5.9%	HbA1c 6—7.9%	HbA1c > 8%	p-value
	n = 29	n = 32	n = 36	
Plaque (%)	95.45 ± 21.32	89.69 ± 28.40	92.10 ± 24.36	0.69
Gingival redness (%)	20.45 ± 36.05	9.25 ± 22.72	3.16 ± 9.54	< 0.04*
Bleeding on probing (%)	16.19 ± 24.58	12.96 ± 20.65	18.16 ± 23	0.62
Suppuration (%)	0.19 ± 0.65	0.31 ± 1.04	0.049 ± 0.265	0.17
Pocket probing depth (mm)	1.58 ± 0.65	1.60 ± 0.67	1.54 ± 0.39	0.91
Clinical attachment loss (mm)	2.45 ± 1.03	2.47 ± 1.77	3.66 ± 2.41	< 0.02*

Plaque, gingival redness, bleeding on probing, suppuration, pocket probing depth, clinical attachment loss are show as mean ± SD; all presented

* p values are based on repeated measures ANOVA

### Gelatinolytic activity of MMP-2 and TIMP-1 in WSS

**Fig. 1:**
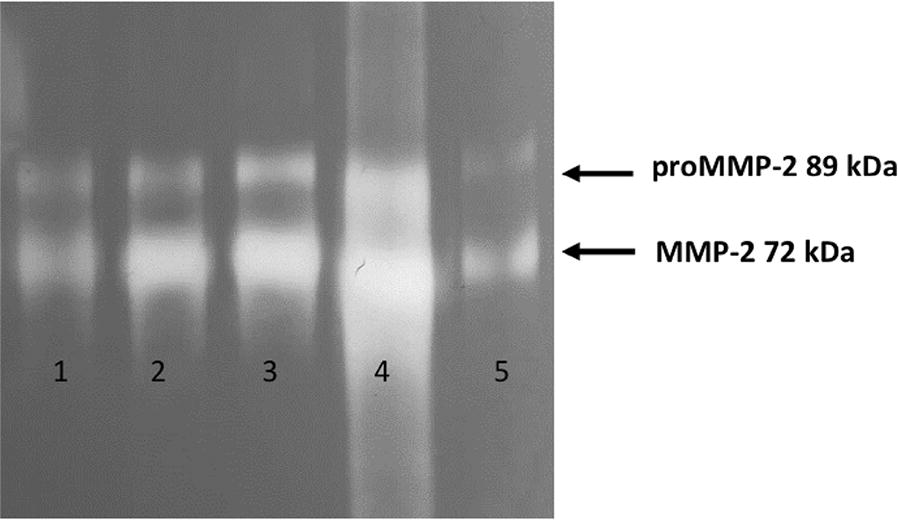
Representative zymography image of gelatinolytic activity of MMP-2 in saliva; lane 1 (Periodontally Healthy), lane 2 (Mild Periodontitis), lane 3 (Moderate Periodontitis), lane 4 (Severe Periodontitis) and lane 5 (MCF7 cell line supernatant positive control)

**Fig. 2:**
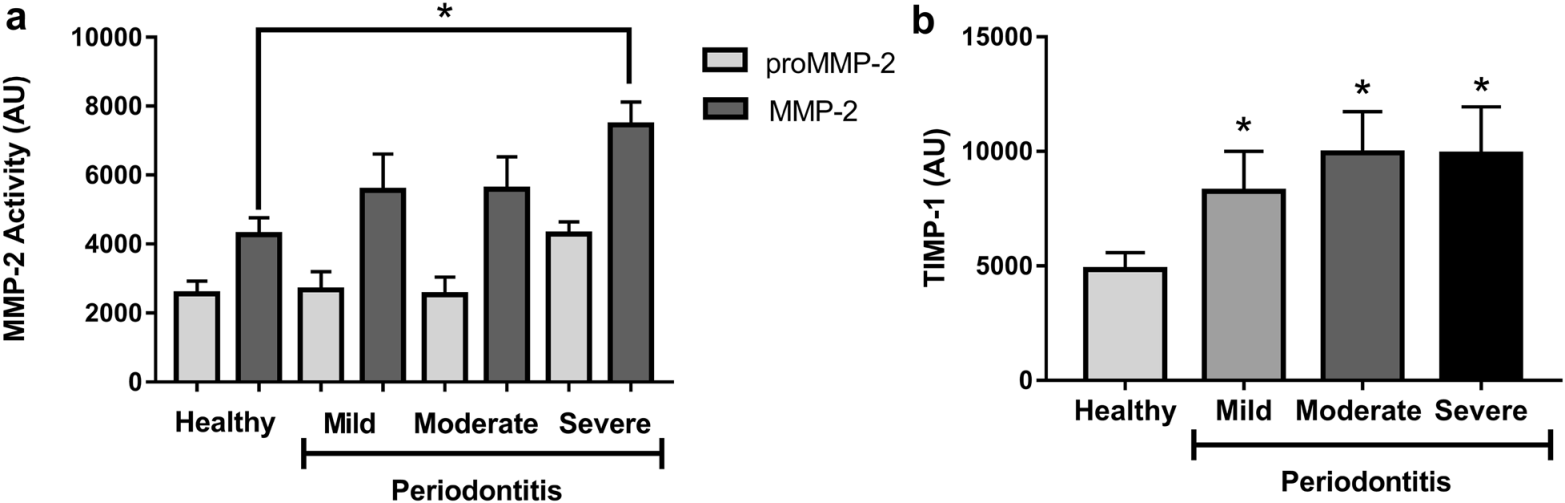
Gelatinolytic activity detected by zymography assay in WSS. **a** Increased MMP-2 gelatinolytic activity was found in Severe Periodontitis in comparison to that of Healthy individuals (*p < 0.05). **b** TIMP-1 were detected by Western blot assay in WSS, and a higher quantity was found in Severe Periodontitis compared to that of healthy individuals (*p < 0.05)

MMP-2 gelatinolytic activity and TIMP-1 protein in WSS were determined as described in “Materials and methods”. A representative zymography gel shows the gelatinase activity of WSS from periodontally healthy individuals, and patients with mild, moderate or severe periodontitis had increased activity of MMP-2 that corresponded with the severity of periodontal disease (Fig. 1). After the zymography assay, the bands relative to the gelatinases were detected; more specifically, bands at the molecular weights 89 kDa and 72 kDa corresponded to pro-MMP-2 and MMP-2, respectively. A significant increase in gelatinolytic activity was found in patients with severe periodontitis compared to that of periodontally healthy individuals (Fig. 2a, p < 0.05). TIMP-1 were significantly higher in patients with mild, moderate and severe periodontitis compared to those of periodontally healthy patients (Fig. 2b, p < 0.05).

### Uncontrolled glycemia affects the gelatinolytic activity of MMP-2 in WSS

**Fig. 3:**
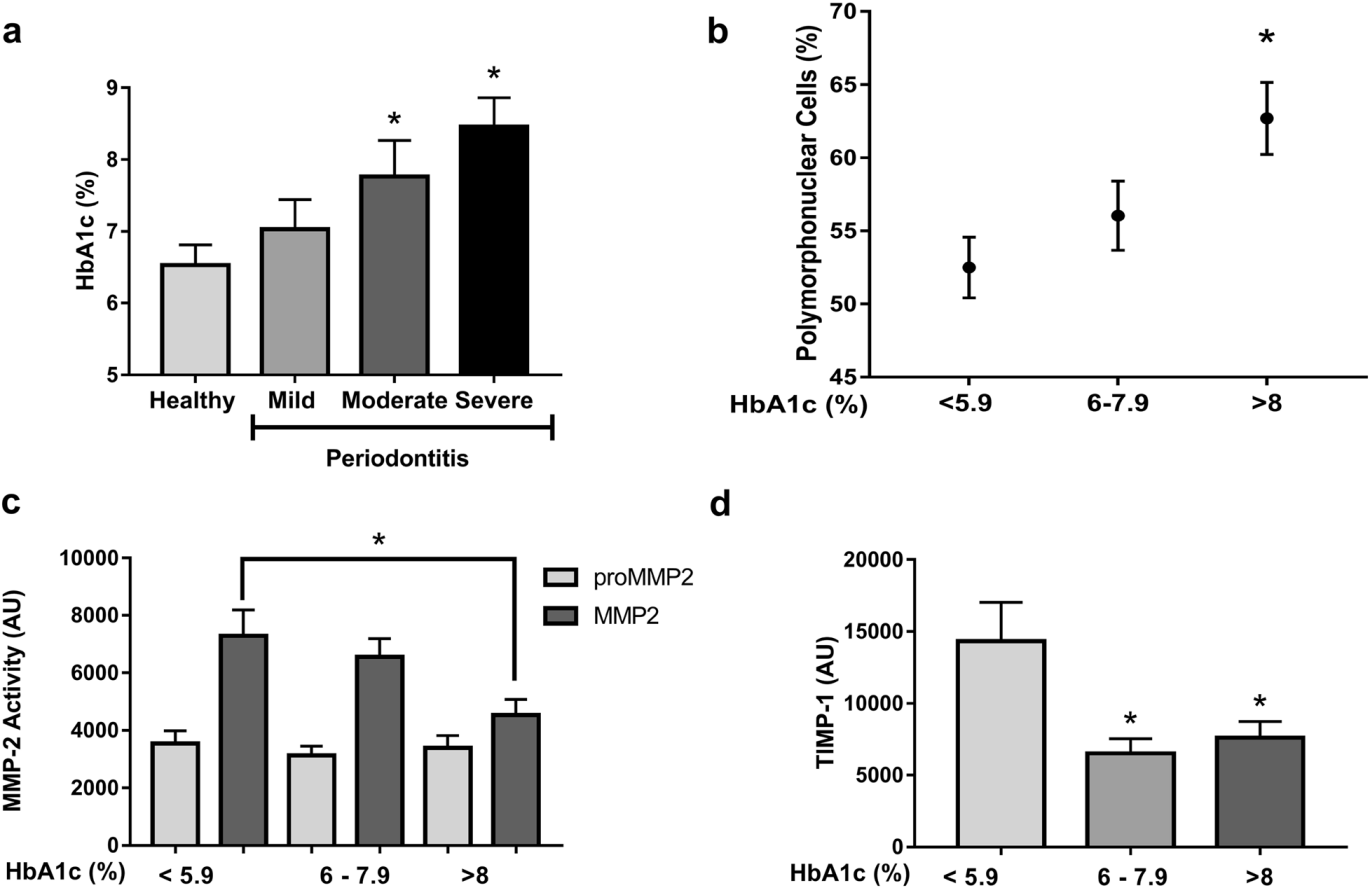
Levels of HbA1c negatively correlate with the activity of MMP-2 and TIMP-1 in WSS. **a** There was a higher percentage of HbA1c in increased Severe Periodontitis in comparison to that of healthy individuals (*p < 0.05). **b** The total count of polymorphonuclear cells increased in patients with HbA1c > 8% compared with that of patients with good control < 5.9% (*p < 0.05). **c** Activity of proMMP2 and MMP-2 decreased in the WSS of patients with a higher percentage of HbA1c (> 8%) compared to that of patients with good glycemic control (< 5.9) *p < 0.05. **d** TIMP-1 in the WSS of patients with T2DM compared with that of patients with different percentages of HbA1c (*p < 0.05)

Based on the percentage of glycosylated hemoglobin, patients were classified as having good (HbA1c ≤ 5.9%), regular (HBA1c 6.0–7.9%) or poor (HBA1c > 8%) glycemic control. A significant increase in the levels of HbA1c in patients with moderate and severe periodontitis compared with that of patients with periodontal health was found (Fig. 3a, p < 0.05). Moreover, polymorphonuclear cell counts were higher in patients with poor glycemic control compared with those of patients with good glycemic control (Fig. 3b, p < 0.05). In contrast, patients with poor control of HbA1c levels showed a lower gelatinolytic MMP-2 activity compared with good control (Fig. 3c, p < 0.05). Moreover, patients with regular and poor control of HbA1c showed the lower quantity of TIMP-1 compared with good glycemic control (Fig. 3d, p < 0.05).

### Uncontrolled glycemia increases the release of nitric oxide in WSS

**Fig. 4:**
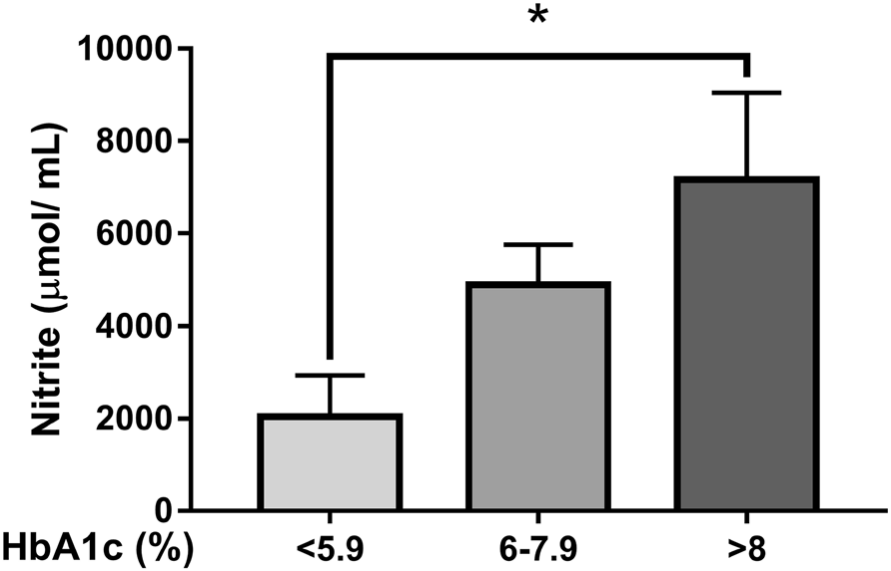
Levels of nitrite in WSS correlate with HbA1c. Micromolar concentrations of nitrite were calculated from the standard curve constructed with sodium nitrite as a reference compound. The data are representative of three independent experiments; (*p < 0.05) significantly different between poor glycemic control (> 8% HbA1c) and good glycemic control (< 5.9% HbA1c)

The concentration of nitrite was evaluated in WSS from patients with T2DM who were grouped according to the percentage of glycosylated hemoglobin. The patients were classified as having a good (HbA1c ≤ 5.9%), regular (HBA1c 6.0–7.9%) or poor (HBA1c > 8%) glycemic control. A significant increase in nitrite concentration was found in patients with poor glycemic control compared to that of patients with good glycemic control (Fig. 4, p < 0.05).

## Discussion

The current study compared and analyzed the periodontal status, HbA1c levels, MMP-2 activity, TIMP-1 and percentage of polymorphonuclear cells in T2DM patients. Clinical periodontal evaluation showed significantly more sites with pocket probing depth, clinical attachment loss, and reduced activity of MMP-2, and these were correlated with the increased levels of glucose in T2DM. This study also suggests a sequence of events resulting from uncontrolled glycemic-dependent periodontal abnormalities leading to microvascular complications in periodontal tissue due to an imbalance between MMP-2 and TIMP-1.

In the clinical periodontal evaluation in the present study, T2DM patients with poor glycemic control exhibited significantly fewer sites with gingival redness and more sites with clinical attachment loss. Moreover, in previous studies, bleeding on probing and suppuration were indicators of periodontal disease risk and severity in T2DM [5, 37]. The damage to periodontal tissue due to uncontrolled glycemic is caused by thickening of the capillary basement membrane, and the appearance of microaneurysms are a morphological hallmark of diabetic microvascular complications [38]. These results suggest that poor glycemic control could develop changes morphological in periodontal tissue which increases the possibility to trigger periodontitis in T2DM.

The increase levels of glucose in the blood and changes in inflammatory molecules cause alterations in the periodontal tissue [36], thereby modulating the activation of different kinases and proteases involved in the development of periodontitis [16]. Recent studies have demonstrated an association between MMP-2 and periodontitis in patients without diabetes [9, 11, 12, 19, 39]. Nevertheless, the relationship between periodontitis and the gelatinolytic activity of MMP-2 in T2DM patients is contradictory [23, 24]. In the present study, MMP-2 showed increased activity in severe periodontitis compared to periodontally healthy, but in mild and moderate periodontitis did not differ significantly from the healthy group. Moreover, TIMP-1 values were elevated in these lower stages of periodontitis severity. These results suggest that the process of inhibition of MMP-2 activity by TIMP-1 is working in mild and moderate periodontitis, being lost in severe periodontitis by other factors which could be the chronic glycemic uncontrol into development the periodontitis in T2DM.

T2DM and periodontitis have recently been found to have a bidirectional relationship [8] in which the presence of periodontitis negatively affects glycemic control and the presence of diabetes increases the risk of developing periodontitis, including abnormalities that involve multiple and synergistic adverse events on the severity of periodontal disease such as advanced glycation end-products [40], polymorphonuclear dysfunctions [41], increased inflammation [42], oxidative stress [43] and loss of blood supply [44]. In the present study, the number of polymorphonuclear cells increased with poor glycemic control, contrary to the decrease in MMP-2 activity in poor control and TIMP-1 in patients with regular and poor control. These results suggest that poor control glycemic in T2DM patients generates increased levels of polymorphonuclear in the blood, but those cells lack the ability to defend against and the activity of MMP-2/TIMP-1, where TIMP-1 is more susceptible to a change in glycemic control in periodontal tissue, developing an unbalance in relating MMP-2/TIMP-1 to develop periodontitis [45, 46].

Oxidative stress is the basis of T2DM, chronic inflammation and how it modifies the pathogenesis of different diseases are currently not well understood [47]. Several studies have found that oxidative stress in T2DM increases salivary reactive oxygen species, lipid peroxidation, nitric oxide, and nitrites [48–50]. Our results showed an increasing concentration of nitrites in WSS from patients with poor glycemic control. These findings might be due to chronic stress caused by high glucose levels in the blood and could be a factor promoting the development of periodontitis.

## Conclusions

In conclusion, in T2DM with glycemic uncontrol conditions, distinct clinical alterations in periodontal tissue were identified, including a decreased in the gingival redness, increased the clinical attachment loss and imbalance of MMP-2/TIMP-1, as the possible cause of disorders promoting the progression of periodontitis. Accelerated periodontitis development in T2DM with uncontrol glycemic likely results from the altered response of host defenses and decreased activity of polymorphonuclear cells. Therefore, these findings identify MMP-2 as a promising molecular marker for periodontitis.

## Data Availability

All data generated and/or analyzed during this study is available from the corresponding author upon reasonable request.
